# TME15/458: Next Generation Telemedicine Network Service for Counselling on Diagnosis of Pigmented Skin Tumours at the Point of Care

**DOI:** 10.2196/jmir.1.suppl1.e122

**Published:** 1999-09-19

**Authors:** A Orel, T Gornik, HP Soyer, I Bartenjev I

**Affiliations:** ^1^Marand D.O.O, LjubljanaSlovenia

**Keywords:** Image Interpretation, Computer-Assisted, Diagnosis, Decision Making, Remote Consultation

## Abstract

**Introduction:**

It is estimated that one in every 70 persons born in 2000 will develop malignant melanoma, one of the most deadly forms of cancer, and that the number of cases doubles every 6 years. The progressing sub-specialisation in medicine results in concentration of experts in few medical centres and deficits of expert knowledge in other, predominantly rural areas. The patient has to be sent to an expert in a medical centre for further diagnosis, which means inconvenience for the patient and costs for the medical care system. Contact between physicians and specialists will be strengthened and the transfer of medical knowledge from centres to periphery intensified.

**Methods:**

Teledermoscopy, the combined use of dermoscopy and telemedicine technologies represents a new approach for the diagnosis of pigmented skin tumours including malignant melanomas. Value of new internet technologies to examine the skin of patients with digital cameras, sending the pictures to centres of excellence for diagnoses and returning them to the points of care will be proven. The possibility of the immediate second opinion on diagnoses is a major advantage of this network.

**Results:**

A network infrastructure, consisting of computer systems, software and communications equipment to support teledermoscopy services will be installed. Applications based on expertise in medicine as well as information technologies will be developed, allowing multi-lingual, user-friendly and secure access to the network from any computer connected to the Internet. Examination of patients will be performed on a face-to-face basis in centres of excellence and by about thirty general physicians and dermatologists. For the purpose of dermatalogic diagnostics different digital cameras will be evaluated. Digital images will be electronically submitted to the TELDOM Platform, a multi-tier, CORBA-based distributed application framework. An automated system of image analysis in order to analyse size, structure and colours of the lesions together with a diagnostic algorithm using artificial networking techniques will be developed.

**Discussion:**

The internet based network will develop an innovative medical system for screening, diagnosis and therapy of skin tumours. It will improve quality of life by providing a user-friendly society and thus increase quality of health by providing easy access to expert medical counselling for individuals with equivocal pigmented skin tumours which will be realised with new generation telemedicine systems for teleconsultation and telecare at the point of need.

